# Local Arctic air pollution: Sources and impacts

**DOI:** 10.1007/s13280-017-0962-2

**Published:** 2017-10-26

**Authors:** Kathy S. Law, Anke Roiger, Jennie L. Thomas, Louis Marelle, Jean-Christophe Raut, Stig Dalsøren, Jan Fuglestvedt, Paolo Tuccella, Bernadett Weinzierl, Hans Schlager

**Affiliations:** 1 LATMOS/IPSL, UPMC Univ. Paris 06 Sorbonne Universités, UVSQ, CNRS, Paris, France; 2 LATMOS/UPMC Univ Paris 06 Sorbonne Universités, Paris, France; 3 Present Address: CICERO, Gaustadalléen 21, Oslo, Norway; 4 Department of Physical and Chemical Sciences, University of L’Aquila, L’Aquila, Italy; 5 Institute of Atmospheric Physics, DLR Oberpfaffenhofen, Wessling, Germany; 6 Boltzmanngasse 5, 1090 Vienna, Austria

**Keywords:** Air pollution, Arctic, Climate, Human health

## Abstract

Local emissions of Arctic air pollutants and their impacts on climate, ecosystems and health are poorly understood. Future increases due to Arctic warming or economic drivers may put additional pressures on the fragile Arctic environment already affected by mid-latitude air pollution. Aircraft data were collected, for the first time, downwind of shipping and petroleum extraction facilities in the European Arctic. Data analysis reveals discrepancies compared to commonly used emission inventories, highlighting missing emissions (e.g. drilling rigs) and the intermittent nature of certain emissions (e.g. flaring, shipping). Present-day shipping/petroleum extraction emissions already appear to be impacting pollutant (ozone, aerosols) levels along the Norwegian coast and are estimated to cool and warm the Arctic climate, respectively. Future increases in shipping may lead to short-term (long-term) warming (cooling) due to reduced sulphur (CO_2_) emissions, and be detrimental to regional air quality (ozone). Further quantification of local Arctic emission impacts is needed.

## Introduction

Air pollution in the Arctic can have adverse effects on climate, ecosystems and health. Whilst air pollutants originate primarily from mid-latitude anthropogenic emission regions in Asia, Europe and North America or from boreal or agricultural fires (Law et al. 2014), sources of local pollution are already known to be important but their emissions and impacts are poorly quantified. In addition, Arctic warming, combined with favourable economic conditions, may lead to further industrial development in the Arctic. This includes increasing possibilities for transport of goods via northern sea routes, resource extraction and associated infrastructure developments and urbanisation. Growth in such activities is likely to increase emissions of air pollutants and add to pollutant burdens in the Arctic.

Air pollutants include trace gases such as ozone [a secondary pollutant formed in the presence of nitrogen oxides (NO_*x*_) and hydrocarbons, including volatile organic compounds (VOCs), carbon monoxide (CO) and methane], or aerosols such as black carbon (BC) or sulphate [formed from sulphur dioxide (SO_2_) emissions]. They are responsible for poor air quality and detrimental effects on human health even at low concentrations (European Environment Agency, EEA 2015). Air quality guidelines set thresholds for ozone and, in the case of aerosols, for particulate matter (e.g. PM_2.5_—sum of particle mass concentrations with an aerodynamic diameter less than 2.5 μm). Pollutants can also cause damage to ecosystems via deposition onto, for example, forests and crops, and impact climate by warming (e.g. ozone, BC) or cooling (e.g. sulphate) the atmosphere. BC can be deposited onto snow and ice surfaces decreasing surface albedo leading to additional warming. Reducing targeted emissions of the so-called short-lived climate forcers (BC, methane which is also an important ozone precursor), in addition to carbon dioxide (CO_2_) reductions (which is very long-lived and often co-emitted), has received much attention due to the potential co-benefits of improving air quality and slowing global/Arctic warming (e.g. Arctic Monitoring and Assessment Programme, AMAP 2015).

Whilst much attention in recent years has focused on improving the understanding about remote sources of Arctic air pollution transported from mid-latitudes and their impacts on climate, rather little attention has been paid to improving our understanding about local emissions and their impacts on climate, ecosystems and health. Earlier studies identified the existence of emissions within the Arctic such as sulphur containing pollution from metal smelting, for example in the Kola Peninsula (Russia), as a source of Arctic sulphate aerosols (e.g. Prank et al. 2010). More recently, Stohl et al. (2013), using ECLIPSE (evaluating the climate and air quality impacts of short-lived pollutants) emissions identified flaring associated with oil/gas extraction in northern Russia and seasonally varying domestic wood combustion as important sources of Arctic BC.

Previous studies also examined the effects of Arctic shipping on present-day and future atmospheric composition (Granier et al. 2006) and deposition of acidic compounds (nitrate, sulphate) in some cases leading to exceedances in critical loads (Dalsøren et al. 2007). Corbett et al. (2010) developed ship emission scenarios, taking into account future growth in shipping, emission regulations [e.g. International Maritime Organisation (IMO) sulphur reductions in ship fuel, reduced NO_*x*_ emissions due to improvements in ship engines] and shipping diverted from southerly routes. Building on Corbett et al. (2010), Winther et al. (2014) developed new inventories, making use of high-resolution automatic identification system (AIS) satellite position data, and predicted modest increases in ozone (> 10%) and large increases in BC (> 80%) along Arctic diversion shipping routes in 2050.

This paper summarises the main results from the European Union ACCESS (Arctic Climate Change, Economy and Society) project (2011–2015) (see Crépin et al. 2017) aiming to improve characterisation of shipping and petroleum extraction emissions in the Arctic and their impacts on atmospheric composition, regional air quality and climate. Since characterisation of these emissions and their impacts is limited by a lack of in situ data collected in close proximity to sources under Arctic conditions (e.g. stable boundary layers, cold temperatures), new aircraft data were collected along the Norwegian coastal region, and used, together with modelling, to examine the impacts of local emissions on Arctic atmospheric composition and climate in the European Arctic. In addition, as part of ACCESS, present-day and future impacts of local pollution on climate, as well as potential impacts on regional air quality, were estimated over the Arctic using regional and global modelling. Global modelling studies were carried out in collaboration with the Norwegian project ArcAct (unlocking the Arctic Ocean: the climate impact of increased shipping and petroleum activities) (2012).

We first present the overall research objectives together with a description of the tools that were deployed, and then describe the main findings about local Arctic pollution emissions and their impacts on atmospheric composition, regional air quality and climate. Finally, we present conclusions and future perspectives.

## Objectives and methodology

As part of ACCESS, the main objectives related to air pollution were to:

1. Derive, for the first time, independent estimates of air pollutant emissions related to Arctic shipping and oil and gas extraction activities under Arctic conditions, with a focus on the Norwegian coastal region;
2. Better quantify the impact of current and future Arctic shipping and oil/gas extraction activities on Arctic chemical composition, climate and regional air quality in the European Arctic and over the entire Arctic region.

To achieve these objectives, we used a combination of new aircraft observations, data analysis, pollutant dispersion and regional/global chemical–aerosol–climate modelling. The main approaches that were deployed are briefly described below.

### ACCESS airborne campaign

**Fig. 1:**
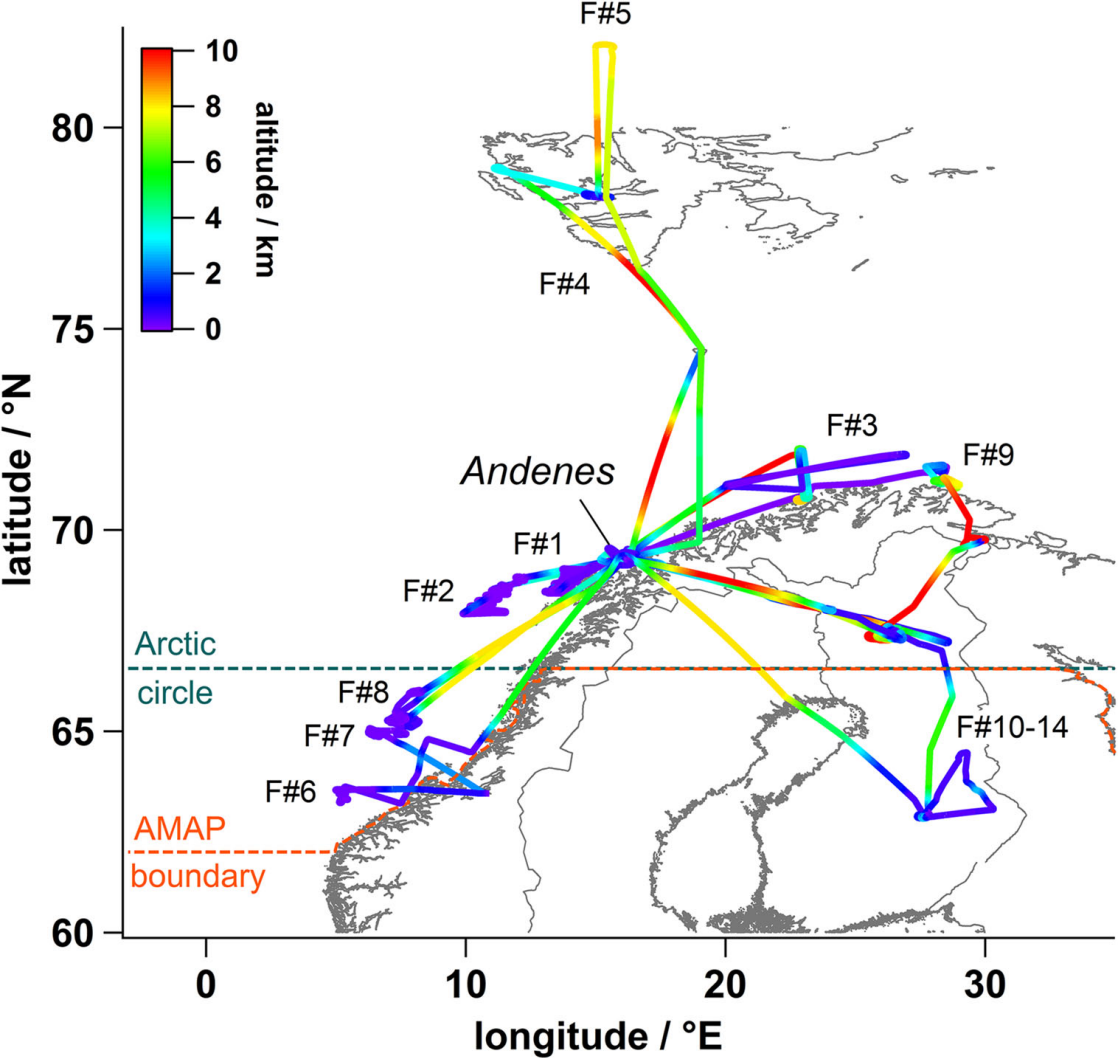
Map showing the DLR-Falcon aircraft flight tracks during the ACCESS airborne campaign based in Andenes (69.29N, 16.14E), northern Norway, in July 2012. Flights focused on sampling emissions from shipping (#1, 2, 6, 9), oil/gas extraction activities (#7, 8), Kola Peninsula metal smelting (#3) and Siberian biomass burning (#4, 5). Flights #10–14 were survey flights over northern Scandinavia (see Roiger et al. 2015). Flight altitudes are indicated on the colour scale. The AMAP region (orange) and the Arctic Circle (turquoise) are also indicated. From Roiger et al. (2015). ©American Meteorological Society. Used with permission

The ACCESS aircraft campaign, using the Deutsches Zentrum für Luft- und Raumfahrt (DLR) Falcon-20 research aircraft, was successfully conducted based in Andenes, northern Norway, from 9 to 27 July 2012, with a total of 9 out of 14 flights sampling local Arctic emissions. Figure 1 shows the flight tracks from the campaign coloured by altitude. Full details about the campaign, including the instrument payload, the campaign design and flight planning (meteorological and tracer forecasts), the flights, meteorological conditions, air masses that were sampled, are given in Roiger et al. (2015). Local pollution plumes were sampled in the vicinity of oil and gas platforms in the southern Norwegian Sea as well as behind different types of ship in Arctic waters along the western and northern Norwegian coast. The flights took place within the Arctic region defined by the Arctic Council’s AMAP. Air masses originating from Russian smelting (Kola Peninsula), and Siberian boreal fire emissions containing enhanced levels of pollutants were also sampled. Measurements included meteorological variables, trace gases (NO_*x*_, SO_2_, CO, ozone) and aerosol instrumentation to measure particle number/size distributions. BC was observed as accumulation mode refractory BC (rBC) mass mixing ratios using a single particle soot photometer (SP2). Pollutant plumes from shipping and petroleum extraction were sampled by making multiple plume crossings at different distances from sources at different altitudes. This approach was chosen in order to gain insights into pollutant plume dispersion and processing in the Arctic boundary layer.

### Modelling tools

A variety of models were used in ACCESS for flight planning, data analysis and to assess impacts of Arctic pollution on atmospheric composition and climate.

*WRF*-*Chem* [weather research and forecasting (WRF) mesoscale weather model, including chemistry] is a regional chemical–aerosol transport model. Model simulations, nudged using National Centres for Environmental Prediction final (FNL) meteorological analyses and including detailed chemical and aerosol schemes, were carried out at high resolution (2–3 km up to 15 km) to analyse aircraft data collected in the vicinity of local sources to validate emission inventories and examine the impacts of pollution on atmospheric composition from oil/gas extraction and shipping along the Norwegian coast (Marelle et al. 2016; Tuccella et al. 2017). Runs at lower resolution (100 km) were used to examine the relative contributions of local versus remote mid-latitude sources of emissions (Marelle 2016).

*OsloCTM2* is a global chemistry transport model (CTM) for the troposphere and stratosphere run at T42 resolution (2.8 × 2.8°) with 60 vertical layers using meteorological data from the integrated forecast system (IFS) model from the European Centre for Medium-Range Weather Forecasts (Dalsøren et al. 2013). A tropospheric version of the model was used including tropospheric chemistry and sulphate, primary organic, nitrate and sea salt aerosols.

*Radiative forcing* (*RF*) *model* calculations were performed using a radiative transfer model (Myhre et al. 2009) developed from the DISORT code-base with four short-wave radiation bands and eight angular multiple scattering streams. Temporal and spatial resolutions were the same as OsloCTM2.

## Results

In the following sections, we describe the principal results from ACCESS related to evaluation of emission estimates and quantification of impacts of local Arctic pollution on climate and air quality at regional (European Arctic) and Arctic-wide scales.

### Improved estimation of local Arctic emissions

**Fig. 2:**
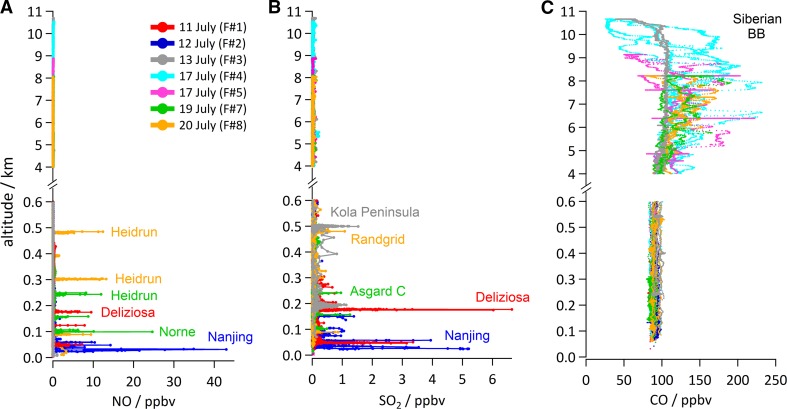
ACCESS aircraft campaign data for **A** NO, **B** SO_2_ and **C** CO showing vertical profiles from different flights. Very high enhancements in emissions can be seen at low altitudes due to sampling of pollutant plumes in the vicinity of oil/gas facilities (e.g. Heidrun, Norne, Asgard C, Rangrid) and shipping (Deliziosa, Wilson Nanjing). Aged boreal fire plumes from Siberia were sampled at higher altitudes with enhanced CO concentrations as well as one plume at lower altitudes from the metal smelting region in north-west Russia (Kola). From Roiger et al. (2015). ©American Meteorological Society. Used with permission

ACCESS aircraft campaign data provided valuable new insights into atmospheric compounds emitted from shipping and oil/gas extraction under summertime Arctic conditions and was used to validate recent emission inventories. Figure 2 shows plume snapshots for pollutants in the Arctic lower troposphere (below 600 m). It can be seen that significant local enhancements in nitrogen oxide (NO) and SO_2_ are present in cases when pollution from shipping, petroleum extraction or metal smelting activities were sampled downwind of the emission source (0–50 km). These elevated plumes mix into background air which may also be impacted by pollution transported from mid-latitudes. During the campaign, CO was enhanced in the middle and upper troposphere (5–10 km; Fig. 2) due to long-range transport of CO from fires in Siberia (Roiger et al. 2015; Raut et al. 2017). CO was not enhanced in the plume samplings since it is not emitted in large quantities from these local sources. Accumulation mode rBC was also observed during the campaign, albeit at rather low concentrations in the fire plumes (Raut et al. 2017) as well as in plumes downwind of certain oil/gas extraction facilities and occasionally in ship plumes (Roiger et al. 2015). However, as noted previously by other studies (e.g. Buffaloe et al. 2014), observations of rBC close to emission sources were often near the lower SP2 cut-off diameter (80 nm), with many very small particles beyond this lower size range being present in fresh plumes, making it difficult to use the SP2 data to estimate emissions (Roiger et al. 2015).

#### Ship emissions

During flights focusing on ship emissions (Fig. 2), plumes from four ships running on diesel fuel were sampled including a cargo ship, a bulk carrier and a passenger cruise ship run on heavy fuel oil emitting high NO_*x*_, SO_2_ and particles. A cargo ship running on marine gas oil with lower sulphur fuel content producing lower SO_2_ emissions was also sampled (Roiger et al. 2015; Marelle et al. 2016). Fishing ships, also running on diesel fuel, which were not included in previous inventories, such as Corbett et al. (2010), were also sampled for the first time in the Norwegian and Barents Sea showing very variable emissions but generally low SO_2_ and high particle number (two cases presented in Roiger et al. 2015). Marelle et al. (2016) compared plume samplings for four ships to plume dispersion simulations, performed with the Lagrangian particle dispersion model FLEXPART-WRF (Brioude et al. 2013) to derive independent estimates of NO_*x*_ and SO_2_ emission fluxes. These estimates were used to validate emissions for sampled ships from a high-resolution inventory—ship traffic emissions assessment model, version 2 (STEAM2) based on AIS data and taking into account individual ship characteristics (e.g. speed, engine/fuel type) (Jalkanen et al. 2012). Overall, STEAM2 NO_*x*_ emissions were biased high (four cases) and SO_2_ emissions both low and high (two cases) (see Table 4 in Marelle et al. 2016). Large biases in emission estimates by STEAM2 were attributed to incomplete technical data or implementation of emission reduction technology not yet taken into account in STEAM2.

At the same time, the regional WRF-Chem model was run with and without STEAM2 emissions for July 2012 and compared to average vertical profile data from the ACCESS airborne campaign. Whilst large biases were found in STEAM2 compared to the independent estimates for individual ships, the regional model results show better overall agreement with the observations when ship emissions are included. This suggests that STEAM2 reproduces aggregated shipping emissions along the Norwegian coast in summer. Marelle et al. (2016) also note that both STEAM2 ship emission estimates, as well as Arctic-wide emissions from Winther et al. (2014) for 2012, also based on AIS data, are significantly higher in northern Norway than older inventories (Corbett et al. 2010; Peters et al. 2011). Use of AIS data, growth in emissions, together with the inclusion of fishing ships, and more detailed emission calculations, may explain these differences. Arctic focused estimates of ship emissions, taking into account, for example, navigation in sea-ice, would also improve emission estimates (Schröder et al. 2017). Aliabadi et al. (2016) already noted that emission factors, determined from observations behind a research vessel operating in the North-West passage (Canadian Arctic), are sensitive to sea-ice presence, showing, for example, increased NO_*x*_ emission factors when the ship was ice breaking.

#### Petroleum extraction emissions

Aircraft data collected around platforms in the southern Norwegian Sea show a very complex picture with emissions varying between different types of facility (Roiger et al. 2015). Data collected downwind of oil/gas production platforms, operating under normal conditions, showed elevated NO due to power generation, and high numbers of volatile particles but low SO_2_. High numbers of small nucleation mode particles suggest significant new particle production from venting/leaks of VOCs. Particles (and occasionally rBC) and NO were enhanced in plumes downwind of certain installations that were flaring but emissions were very intermittent making it difficult to draw conclusions about these emissions. In contrast, emissions from storage tankers or drilling rigs (also considered as mobile “platforms”) which are essentially stationary ships, as well as shuttle tankers, exhibited high emissions of SO_2_ and associated non-volatile particles (i.e. sulphate), together with high NO due to diesel combustion. Measured rBC showed enhancements downwind of mobile platforms, a potentially more important source than emissions from production platforms (Roiger et al. 2015), but, as noted earlier, instrument cut-off issues in fresh plumes meant it was not possible to characterise these emissions. Overall, measured plumes associated with oil/gas extraction were of similar magnitude compared to ship plumes in the case of NO, but tended to be smaller in the case of SO_2_ (see Fig. 2).

The ACCESS data were compared to WRF-Chem simulations run at high resolution (2 km) in the region of the platforms for 19/20 July 2012 using two inventories providing emissions for specific facilities: TNO-MACC emissions for 2009 and Norwegian Environment Agency (NEA) 2012 emissions (see Tuccella et al. 2017 for details). It is important to note that emissions from mobile platforms, such as certain storage tankers and drilling rigs, are not included in these inventories. In addition, NEA does not report aerosol emissions making it necessary to estimate these emissions (using ratios with NO_*x*_) and TNO-MACC only includes emissions of NO_*x*_ and VOCs. While runs using the NEA emissions agreed reasonably well with the measurements, a run with TNO-MACC, which has much lower NO_*x*_ emissions compared to NEA (by factor 20–30), was unable to represent measured composition in the plumes. One difficulty associated with this analysis is the highly variable nature of emissions from oil/gas production which vary significantly depending on operating conditions, flaring, etc. These results highlight deficiencies in current inventories used in global and regional models which do not take into the account the intermittent nature of certain emissions such as flaring (since only annual average emissions are usually provided) and which do not include emissions for certain species or emissions from particular sources (e.g. mobile platforms).

### Local and regional impacts on atmospheric composition and air quality

Influences of local emissions from shipping and petroleum extraction on atmospheric composition have been investigated using the WRF-Chem model for the campaign region in July 2012 and, in the case of shipping, for the entire Arctic. Whilst the campaign region and period are limited in space and time, these results provide first indications about impacts in a region where local emissions are already occurring.

Based on better agreement with ACCESS data collected around the Norwegian oil/gas platforms, the results of the high-resolution model runs (2 km) with the NEA emissions were used to assess, for the first time, potential impacts of these emissions on Arctic atmospheric composition under Arctic conditions (Tuccella et al. 2017). Modest daytime average enhancements in ozone (up to 7% above background of 25–30 ppbv) were predicted in the Arctic boundary layer with larger noontime increases of up to 4 ppbv (15%) around 600 m up to 50 km downwind of the platforms. Small enhancements in PM_2.5_ were also predicted (11% at the surface) with the largest increases in model BC (+ 48% at the surface). Given that emissions from mobile platforms are not included in the TNO-MACC and NEA emissions, our findings are likely to be lower estimates. However, they suggest that petroleum extraction emissions are already impacting the pristine Arctic troposphere. Fully speciated ozone precursor and aerosol emissions are required to improve predictions of these impacts as well as measurements in regions where these emissions are estimated to be larger such as in northern Russia.

**Fig. 3:**
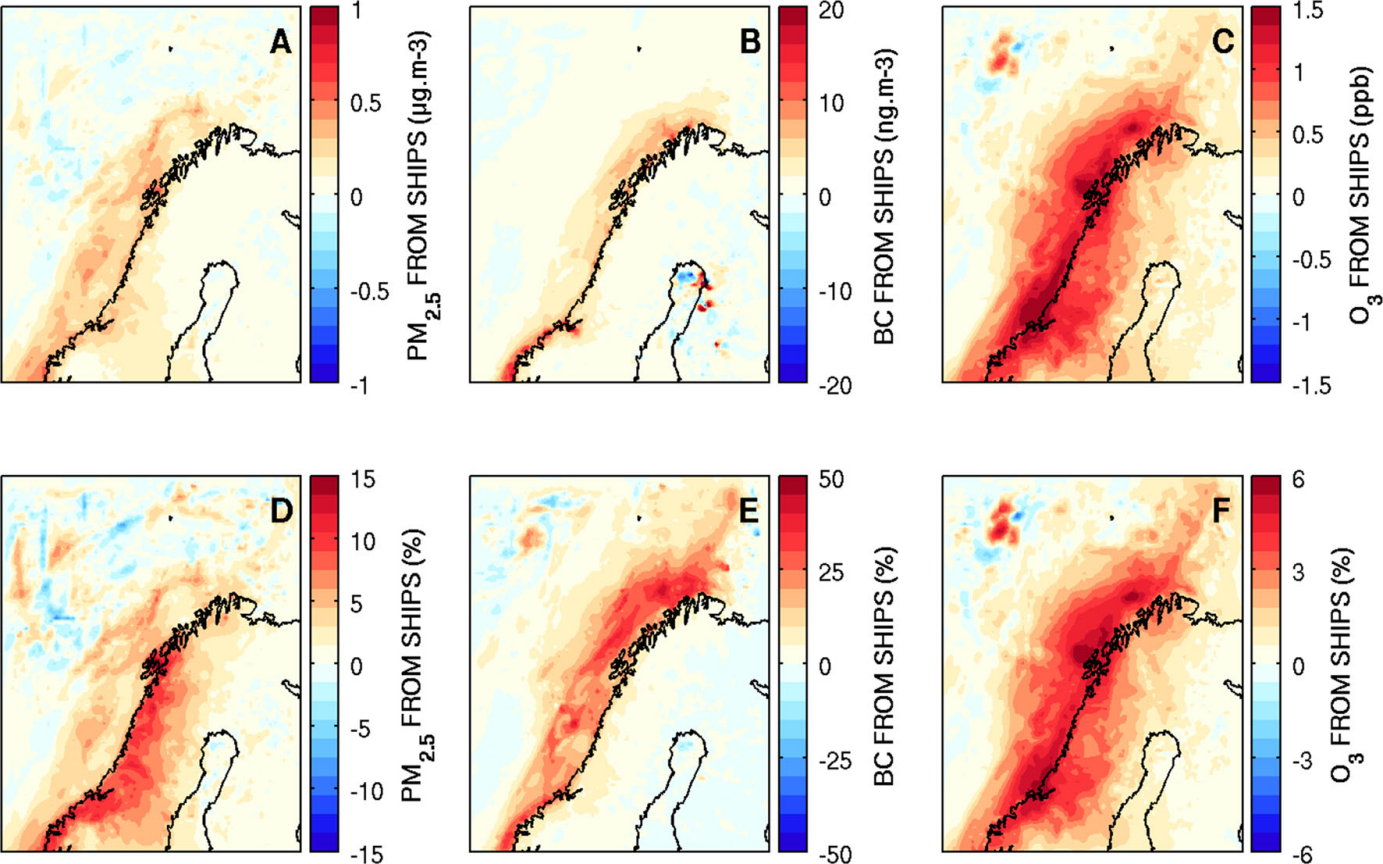
Surface pollution enhancements in particulate matter (PM_2.5_, μg m^−3^), black carbon (ng m^−3^) and ozone (ppb) concentrations and percentages averaged over a 15-day period from 00UT on 11 July 2012 to 00UT on 26 July 2012. Results are based on differences between model simulations run with and without shipping emissions from STEAM2. See text for details. Figures adapted from Marelle et al. (2016)

Results from the WRF-Chem model, run at 15 × 15 km using the STEAM2 ship emissions (re-gridded from individual ship data on 5 × 5 km, and updated every 30 min), show significant enhancements in pollutants due to shipping along the Norwegian coastal region over a 15-day period in July 2012 (Marelle et al. 2016) (see Fig. 3). NO_*x*_ and SO_2_ increased by 80% (average over 15 days), whereas ozone increases were lower (6%, 1.5 ppbv). Marelle et al. (2016) also found small increases in PM_2.5_ of up to 0.5–1.0 μg m^−3^ or about 5–10% above background (5–8.5 μg m^−3^) with main contributions from sulphate (+ 20%) and BC (+ 40%). We note that even small increases in PM_2.5_ could increase concentrations above the annual mean threshold set by the World Health Organisation (WHO) of 10 μg m^−3^. These results show that local Arctic shipping emissions already appear to be influencing Arctic atmospheric composition and potentially air quality in this region. These findings are in agreement with the recent observation-based analyses showing enhancements in equivalent BC due to summertime cruise shipping in Svalbard (Eckhardt et al. 2013), and along the North-West passage (Resolute Bay, Canada) where enhancements in ozone and PM_2.5_ due to ship traffic were observed (Aliabadi et al. 2015).

**Fig. 4:**
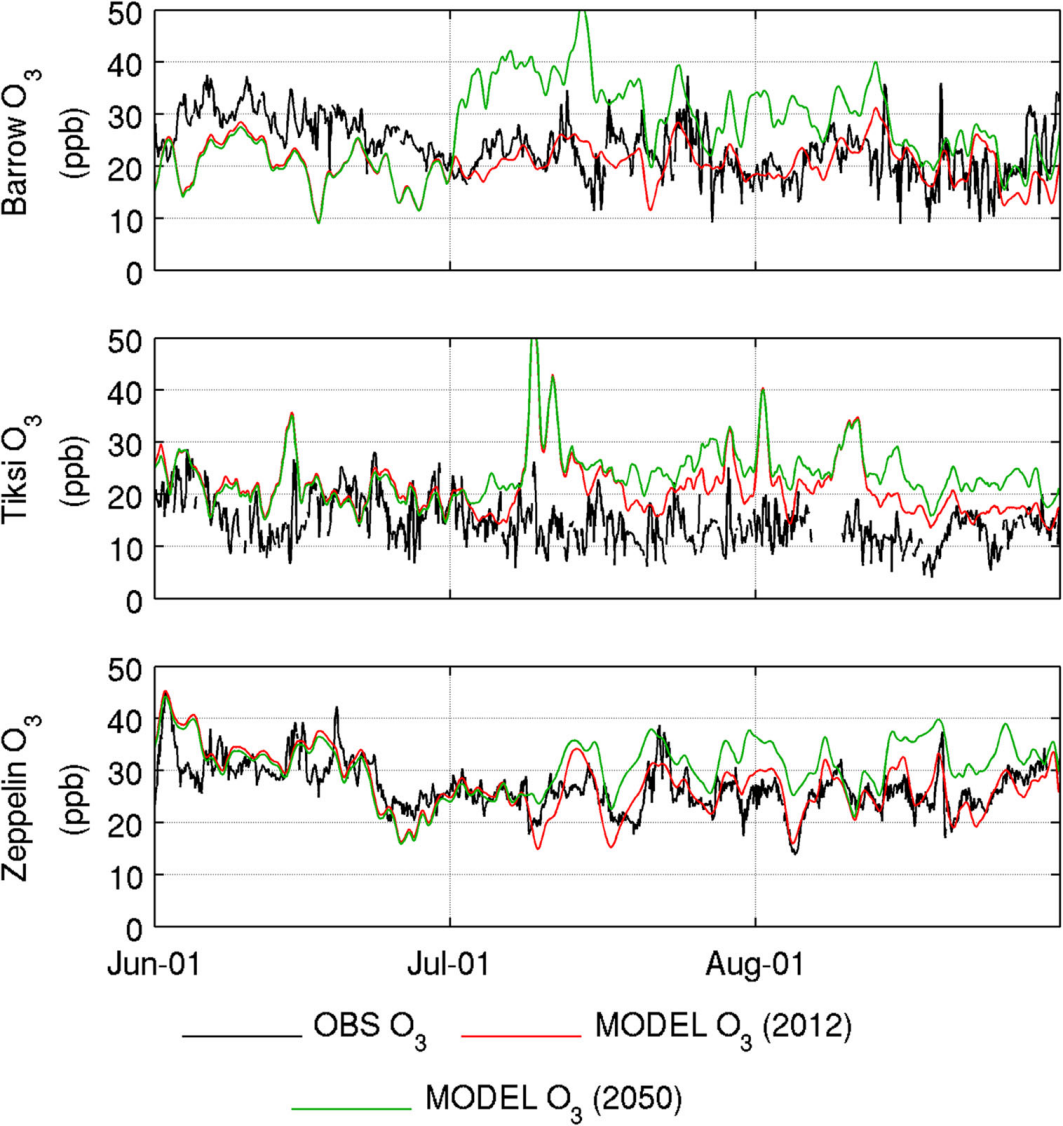
Comparison of results from the WRF-Chem model for present-day (2012) and future (2050) conditions with 2012 observations of ozone at Barrow (Alaska), Tiksi (northern Russia) and Mount Zeppelin (Svalbard). Barrow data are courtesy NOAA-ESRL GMD/PSD, Tiksi data courtesy of Rushydrometeorology/NOAA-ESRL GMD/PSD and Mount Zeppelin data courtesy of NILU. See McClure-Begley et al. (2014) for details about NOAA data. Note that Tiksi ozone data are preliminary due to instrument changes in 2012. Increases in predicted ozone are primarily due to increases in shipping diverted from southerly routes through the Arctic along the North-East and North-West passages during July and August

WRF-Chem has also been run more recently at quasi-hemispheric scales with present-day emissions (2012) and future shipping scenarios (2050) (Marelle 2016). Simulations using ECLIPSE anthropogenic emissions, FINNv2 (Fire Inventory from NCAR, version 2) boreal fire emissions, Arctic shipping emissions (high growth scenario) from Winther et al. (2014) and diversion shipping from Corbett et al. (2010) were used to investigate future shipping impacts on atmospheric composition. Here, we examine potential future impacts of these emissions on local air quality. The results suggest that future shipping may have a significant impact on local air quality along Arctic coastal regions primarily due to increased ozone concentrations. This is illustrated in Fig. 4 showing daily mean model ozone for present-day and future conditions compared to data collected at measurement sites in the Arctic. WHO sets limits for daily 8-h mean ozone concentrations at 50 ppbv although there are suggestions that chronic health impacts may occur at lower concentrations (e.g. http://www.eea.europa.eu/publications/TOP08-98/page010.html). Small increases in PM_2.5_ are also predicted along shipping lanes (up to 2 μg m^−3^). Locally (e.g. port areas), these levels could be exceeded (noting the WHO 24 h limit of 25 μg m^−3^), and depend on full implementation of proposed regulations. NO_*x*_ control via the implementation of nitrogen emission control areas (NECAs) is also being discussed for the North and Baltic Seas. The implementation of such controls in the Arctic requires quantification but could lead to ozone reductions, at least away from shipping lanes. NO_*x*_ reductions in the Arctic may be more effective at reducing ozone since offsetting factors related to ozone production from methane (longer lifetime) are likely to be lower at high latitudes (Jonson et al. 2015).

### Impacts of local emissions on Arctic and global climate

Climate models, including treatments of chemistry and aerosols, were used in ACCESS to quantify the impacts of local emissions on Arctic climate. The results on shipping were summarised in an ACCESS Policy Brief (see http://www.accesseu.org/en/publications/access_brief.html).

**Fig. 5:**
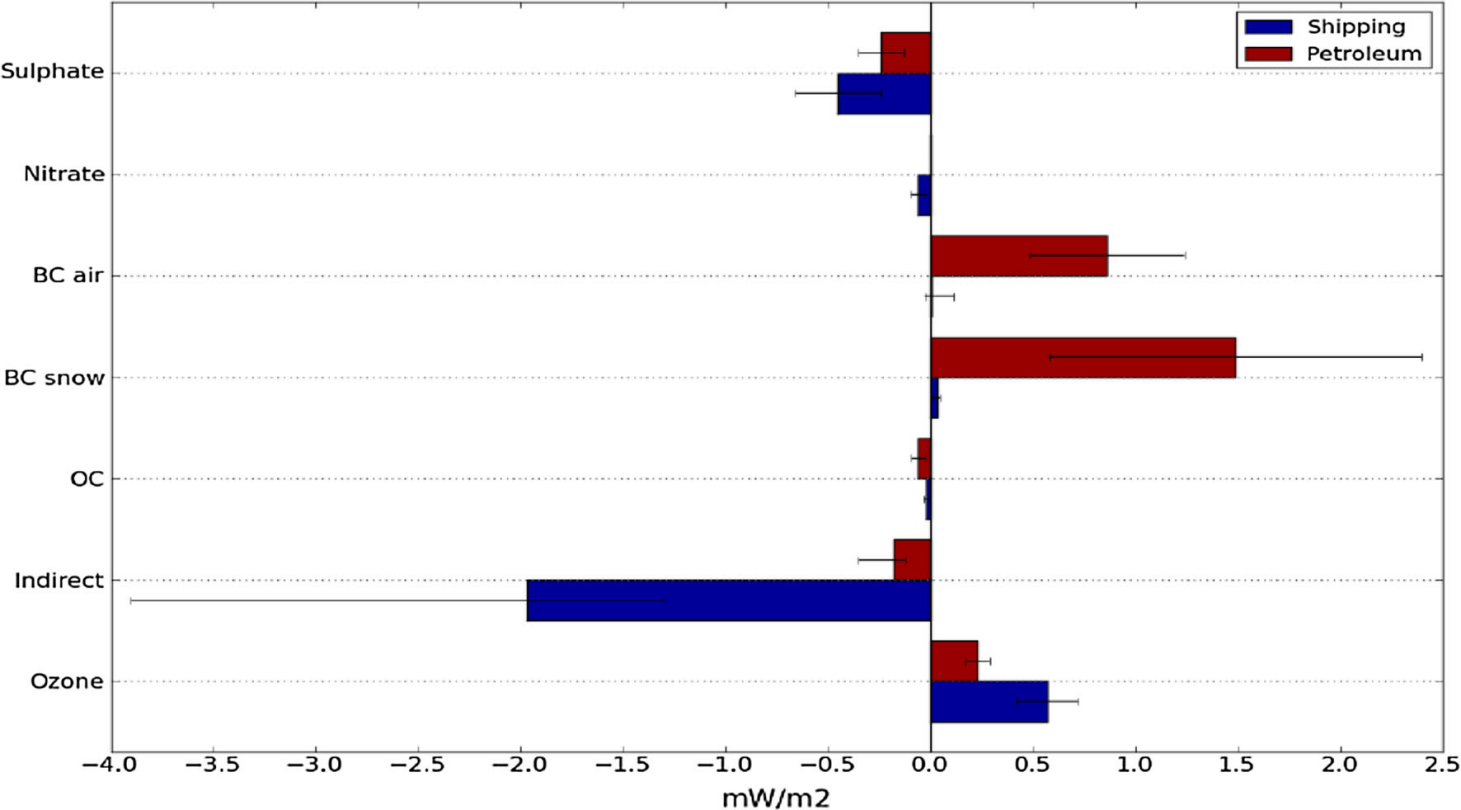
Global and annual radiative forcing (mW m^−2^) for the different pollution components [including aerosol-cloud indirect effects and BC deposition on snow, organic carbon (OC)] from current Arctic shipping and petroleum emissions. Large sulphur emissions result in net negative forcing from shipping. Lower sulphur emissions and higher BC emissions result in net positive forcing from petroleum activities. From Ødemark et al. (2012)

Ødemark et al. (2012) used the OsloCTM2 and RF models to calculate current impacts from petroleum activity and shipping in the Arctic using an emission dataset developed specifically for the Arctic for present-day and future conditions by Peters et al. (2011). They estimated that current petroleum emissions in the Arctic result in a net positive global annual RF (warming) due to low sulphur emissions and high BC emissions (Fig. 5) [+ 20 mW m^−2^ averaged over the Arctic (> 60N)]. In contrast, Ødemark et al. (2012), found that present-day Arctic shipping is causing a net negative global annual RF (cooling), primarily due to direct and indirect radiative effects from sulphate aerosols [− 20 mW m^−2^ averaged over the Arctic (> 60N)]. Results from the WRF-Chem simulations (Marelle et al. 2016) estimated a total negative short-wave forcing just from Norwegian shipping (in July 2012) that is similar to the forcing estimated by Ødemark et al. (2012) (− 10.4 mW m^−2^ global average) suggesting that these effects may be larger than previously estimated. This is likely due to the inclusion of the second indirect effect and the semi-direct effect in Marelle et al. (2016). Assessment of present-day impacts of local emissions are very sensitive to assumptions made about different types of emission. For example, the results from ACCESS have highlighted the intermittent nature of flaring emissions, missing emissions from mobile platforms or certain species in current inventories, and potential formation of organic aerosols from venting which will influence such assessments.

Future climate impacts of short-lived atmospheric pollutants due to global and Arctic shipping emissions were estimated by Dalsøren et al. (2013). OsloCTM2 was run with high growth (HIGH) and maximum feasible reduction (MFR) scenarios for 2030 from Corbett et al. (2010) including moderate to substantial increases in pollutants both globally and in the Arctic (especially in summer). Exceptions are found in the MFR scenario when technological advances are included reducing BC emissions by 70%. Implementation of future IMO regulations reducing fuel sulphur content leads to reductions in emissions of SO_2_ and therefore lower production of sulphate aerosols. In contrast to cooling from present-day shipping, predicted future changes from 2004 to 2030 result in global average net positive RF [+ 53 mW m^−2^ (HIGH); + 73 mW m^−2^ (MFR)] due to less cooling from sulphate aerosols (additional positive RFs from long-lived components N_2_O and CO_2_ were not quantified). In the Arctic, the overall RF for the HIGH scenario is a factor 1.5 larger than for the MFR scenario, opposite to the global picture, due to relatively stronger RF from ozone and BC, and smaller indirect aerosol effects, in the Arctic.

**Fig. 6:**
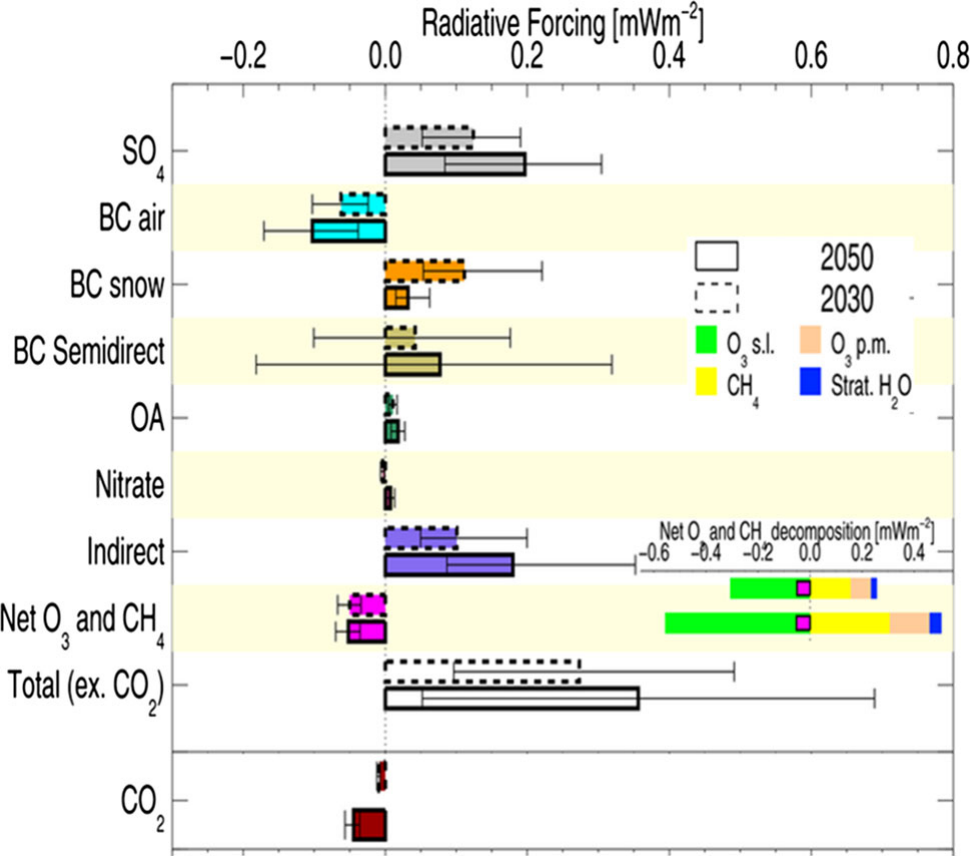
Climate impact in terms of global annual radiative forcing by component for shifting shipping routes from the Suez Canal to the Arctic for 2030 and 2050. Positive global annual radiative forcing results in warming, while negative results in cooling. The bars indicate uncertainty and it is 90% likely that the forcings are within the ranges of the bars. *OA* organic aerosols, *O*_*3*_ *s.l*. short-lived ozone forcing, *O*_*3*_ *p.m*. primary mode ozone forcing (longer lived ozone forcing due to changes in methane which is an important ozone precursor). Adapted from Fuglestvedt et al. (2014)

Whilst these calculations included shipping diverted from southerly routes, Fuglestvedt et al. (2014) investigated this further by examining a shift from the Suez route to the North-East passage showing that transit times are shorter resulting in fuel savings and lower emissions. The shift in shipping leads to global annual average warming from non-CO_2_ components as shown for 2030 and 2050 in Fig. 6. In contrast, the impacts of the long-lived greenhouse gas CO_2_ do not depend on emission location. Net emission reductions result in cooling from CO_2_ which grows over time due to the long response time for CO_2_ (Fig. 6). The net global annual effect from all components (non-CO_2_ + CO_2_) is a warming for the first 150 years, which thereafter switches to cooling. Thus, the possibilities for shifting shipping to the Arctic confront policy makers with the question of how to weigh up century-scale warming with large uncertainties (Fig. 6) versus a long-term climate benefit from CO_2_ reductions.

## Conclusions and perspectives

Results from ACCESS have provided new insights into local Arctic pollution sources and their impacts on climate and regional atmospheric composition/air quality. Aircraft data, and subsequent analyses, have been used to validate current emission inventories for shipping and petroleum-related activities and to assess their impact on atmospheric composition during summertime conditions in the European Arctic. Independent validation of individual sources revealed variable agreement with reported emissions, due to missing sources/species, assumptions about operating conditions or emission control technologies. Current inventories (usually annual means) used by global and regional models need to be improved taking these factors, as well as the intermittent nature of certain emissions (e.g. flaring, shipping), into account. Generally, model results agree better with observations when high-resolution local emissions are included and suggest that ship and oil/gas extraction emissions off the coast of Norway are already having a significant impact on Arctic composition as well as current, and potentially, future air quality. Further characterisation of these and other local emissions (e.g. wood burning, metal smelting) are needed at other times of year, such as winter and spring (when boundary layers are more stable, snow or sea-ice may be present, etc.), to improve assessment of pollutant impacts, not only on climate and regional air quality, but also on ecosystems and human health.

Potential impacts on climate were assessed based on the available scenarios. Present-day shipping and petroleum extraction lead to cooling and warming, respectively, whereas increases in Arctic shipping, as well as shifts from southerly routes, lead to warming in the future, at least in the short term. However, results are very dependent on the employed emission scenarios and how sea-ice melt evolution, economic factors and proposed emission regulations etcetera are taken into account. Reductions in NO_*x*_ emissions (e.g. via NECAs) leading to reductions in ozone, or in BC emissions, would likely benefit air quality and climate, whereas reductions in sulphur emissions from shipping are likely to improve local and regional air quality through PM_2.5_ reductions but warm the climate. This poses a challenge to policy makers. Reducing CO_2_ emissions is the key to an effective climate policy, whereas reductions in air pollutants may either lead to climate warming or cooling. As the Arctic warms, it will be necessary to monitor changes in Arctic composition and take effective mitigation measures. In parallel, further work is still needed to better assess impacts of local pollutants, including contaminants, on human health and to evaluate impacts of pollutant deposition on ecosystems (e.g. nitrate).
